# The effect of ionizing radiation through cardiac stereotactic body radiation therapy on myocardial tissue for refractory ventricular arrhythmias: A review

**DOI:** 10.3389/fcvm.2022.989886

**Published:** 2022-09-15

**Authors:** John Whitaker, Paul C. Zei, Shahreen Ahmad, Steven Niederer, Mark O'Neill, Christopher A. Rinaldi

**Affiliations:** ^1^Brigham and Women's Hospital, Boston, MA, United States; ^2^Harvard Medical Schools, Boston, MA, United States; ^3^School of Biomedical Engineering and Imaging Sciences, King's College, London, United Kingdom; ^4^Guy's and St. Thomas's NHS Foundation Trust, London, United Kingdom

**Keywords:** cardiac stereotactic body radiation therapy, ventricular tachycardia, cellular response, cardiomyopathy, ablation electrophysiology

## Abstract

Cardiac stereotactic body radiation therapy (cSBRT) is a non-invasive treatment modality that has been recently reported as an effective treatment for ventricular arrhythmias refractory to medical therapy and catheter ablation. The approach leverages tools developed and refined in radiation oncology, where experience has been accumulated in the treatment of a wide variety of malignant conditions. However, important differences exist between rapidly dividing malignant tumor cells and fully differentiated myocytes in pathologically remodeled ventricular myocardium, which represent the respective radiation targets. Despite its initial success, little is known about the radiobiology of the anti-arrhythmic effect cSBRT. Pre-clinical data indicates a late fibrotic effect of that appears between 3 and 4 months following cSBRT, which may result in conduction slowing and block. However, there is clear clinical evidence of an anti-arrhythmic effect of cSBRT that precedes the appearance of radiation induced fibrosis for which the mechanism is unclear. In addition, the data to date suggests that even the late anti-arrhythmic effect of cSBRT is not fully attributable to radiation.-induced fibrosis. Pre-clinical data has identified upregulation of proteins expected to result in both increased cell-to-cell coupling and excitability in the early post cSBRT period and demonstrated an associated increase in myocardial conduction velocity. These observations indicate a complex response to radiotherapy and highlight the lack of clarity regarding the different stages of the anti-arrhythmic mechanism of cSBRT. It may be speculated that in the future cSBRT therapy could be planned to deliver both early and late radiation effects titrated to optimize the combined anti-arrhythmic efficacy of the treatment. In addition to these outstanding mechanistic questions, the optimal patient selection, radiation modality, radiation dose and treatment planning strategy are currently being investigated. In this review, we consider the structural and functional effect of radiation on myocardium and the possible anti-arrhythmic mechanisms of cSBRT. Review of the published data highlights the exciting prospects for the development of knowledge and understanding in this area in which so many outstanding questions exist.

## Introduction

The use of single fraction, high dose ionizing radiation therapy (RT) in the form cardiac-stereotactic body radiation therapy (cSBRT) has recently been described as a treatment for patients with refractory ventricular arrhythmias (VA). The clinical experience has been reviewed previously (1) and encouraging results have been reported following the use of cSBRT to treat patients in whom control of VA could not be achieved with conventional therapies. While the clinical response has been encouraging, a precise understanding of the anti-arrhythmic mechanism of cSBRT remains incomplete. A number of observations have been consistently reported that indicate a complex response of myocardium to cSBRT and identify areas in which further work will be required to reach a comprehensive understanding of the mode of action of cSBRT.

Conventional radio-frequency (RF) cardiac ablation comprises thermal injury to myocytes resulting in acute coagulative necrosis and associated with acute cell death and consequent myocardial conduction block (2). Other catheter based ablation energies are also associated with acute cell death and secondary conduction block (3, 4). RT, on the other hand, is not expected to be, and has not typically been demonstrated to be, associated with acute formation of conduction block. Despite this, delivery of cSBRT has been successfully used to achieve acute (within 1 day), as well as chronic, suppression of incessant ventricular arrhythmias (5). This raises the question as to how cSBRT results in early arrhythmia suppression and indicates that in contrast to catheter-based ablation technology, other anti-arrhythmic mechanisms may be important. Following cSBRT at doses that have been delivered clinically, development of fibrosis in the timeframe of months following cSBRT has been reported, at which point ongoing suppression of ventricular arrhythmias with cSBRT has also been reported. This suggests that different mechanisms may be responsible for the acute and chronic phases of the myocardial response to cSBRT. In order to understand how cSBRT may exert its anti-arrhythmic effect, an understanding of the tissue response to RT is paramount. In this review, recent experimental and clinical data relating to the effect of RT on myocardium at different time points following RT exposure is reviewed, and the experimental data regarding the functional response of various myocardial structures is considered. Review of this data raises more questions than it provides answers, reflecting the current status of knowledge in this field. This identifies compelling opportunities for future research to develop our understanding of the mechanisms underlying this promising non-invasive treatment strategy in the field of arrhythmia management.

### Cellular response to ionizing radiation

Radiation induced cellular changes have been studied widely in the field of oncology. The cellular response to radiation has typically been considered with regard to differentiating cells, and interruption of the cell cycle is an important mechanism through which RT affects malignant cells. This represents an important distinction from the situation when RT is used to treat arrhythmias, in which case it is understood that fully differentiated cells, either anatomically selected on the basis of established ablation strategies (for example atrio-ventricular node (AVN), cavo-tricuspid isthmus (CTI) or pulmonary vein (PV) ostia) or abnormal myocardial substrate in the context of ventricular arrhythmias, are targeted. Despite this key difference, there is a wealth of literature relating to the effect of radiation on cells, much of which remains relevant when considering how RT may affect myocardial tissue. The molecular mechanisms underlying radiation induced cellular death have been recently reviewed (6). A number of responses to RT are recognized, including mitotic catastrophe and cell death, apoptosis, necrosis, cellular senescence and autophagy. Broadly, cell death may be considered as regulated (including apoptosis and other less common forms of regulated cell death) or unregulated (necrosis). Cellular senescence describes a condition of permanent cell cycle arrest, and has been associated with a characteristic senescence associated secretory phenotype (SASP) (7). The role of cellular senescence in already cell-cycle arrested cardiomyocytes is incompletely defined, but senescence and the SASP represent relatively recently appreciated contributors to the development of chronic cardiac conditions, in particular those associated with increasing age, and senescence may comprise part of the myocardial response to RT. Mitotic death may be less relevant to the myocardial response to RT than other forms of cell. Apoptosis is a highly regulated process and is associated with characteristic morphologic and molecular features. Specifically, cellular markers may identify activation of apoptotic cellular death pathways. Caspase-3 represents a common marker of multiple pathways of activation of apoptotic cell death (6). In contrast to apoptosis, necrosis represents an unregulated form of cell death that may occur in response to RT induced cellular and micro-environment changes. Necrotic tissue has characteristic morphological features including increased cellular volume, membrane rupture and release of intracellular contents (6). In addition to effecting direct cellular damage, RT may induce secondary cell death. Experimental data from the cancer literature indicates that doses of greater than 10Gy induce severe vascular damage within tumors resulting in hypoperfusion and likely secondary cell death through ischemia, effects which have been observed as early as 24 hours following irradiation (8). It is recognized that neovascularization within tumors renders the blood supply more radiosensitive than surrounding normal tissue (9). RT induced vasculitis represents a potential mechanism through which cSBRT may mediate an ablative effect in myocardium. Secondary effects of RT exposure may also include augmentation of an immune response, which has been suggested in tumor biology. Whether or not a secondary immune response to cSBRT is relevant remains uncertain. It is plausible that there would exist differences in the radiosensitivity of pathological myocardial substrate when compared to adjacent healthy tissue, although evidence to demonstrate this has not been established. Data from experimental and clinical studies following cSBRT have identified features indicating a role for necrosis, apoptosis and vascular injury in the myocardial response to cSBRT and are discussed below.

### Ionizing radiation induced cardiovascular disease

Data from patients undergoing thoracic irradiation for malignancy established the potential for RT to cause cardiovascular damage (10). RT may cause early toxicity such as pericardial inflammation or delayed toxicity affecting the pericardium, valves, conduction system or myocardium (11). The mechanisms underlying RT induced cardiovascular disease (CVD) are relevant when considering the mechanism through which cSBRT may mediate an ablative or anti-arrhythmic effect. Mechanisms through which RT may induce CVD include endothelial damage, through direct DNA damage and oxidative stress, which may result in a greater risk of atherosclerotic plaque rupture. Microvascular obstruction may also occur, which contributes to the development of capillary loss, ischemia, myocardial cell death and subsequent myocardial fibrosis. In addition to promoting vascular effects with a secondary impact on myocardium, direct effects on the myocardium are recognized. Oxidative stress on the cell membrane following RT exposure is a key mechanism underlying the development of myocardial inflammation and subsequent progression to fibrosis, which most commonly manifests as restrictive cardiomyopathy, likely reflecting progressive myocardial fibrosis (12). The generation of reactive oxygen species (ROS) following RT affects mitochondrial function, which represent the primary cellular site of oxidative metabolism (10). RT induced mitochondrial dysfunction promote cellular aging and apoptosis (13). RT induced mitochondrial dysfunction may promote the development of myocardial cellular senescence, and the associated senescence-associated secretory phenotype (SASP) (7). The impact of RT in promoting the SASP in the heart remains incompletely characterized, is likely to be complex and specific to different cell types within the myocardium, but is likely to be relevant to the myocardial response to RT. The QUANTEC project has sought to evaluate the current state of knowledge of the biologic effect of radiation doses on normal tissue (14) and provided data specifically relevant to cardiac toxicity (14). Although the data from the oncologic experience reflects heterogeneous radiation doses and dosing regimes, which in general have been delivered with the aim of minimizing the myocardial dose, the mechanism for myocardial toxicity has been consistently identified as the late development of myocardial fibrosis. This appears to be mediated through vascular as well as direct myocardial effects following an initial inflammatory response to RT.

### Effect of cardiac stereotactic body radiation therapy on myocardial tissue

As the therapeutic potential for cSBRT in the treatment of arrhythmias has become apparent, there has been greater interest in the mechanisms by which high-dose radiation affects myocardium. While data collected from the collateral irradiation of myocardium in cancer patients provides an invaluable foundation for the study of the myocardial response to RT, this data has been collected from studies in which the cardiac dose has been deliberately minimized. More recently, pre-clinical studies and a smaller number of clinical reports have attempted to assess the acute and chronic effect of high-dose single fraction cSBRT on different heart structures. These data are also confounded by the use of different radiation sources and modes of delivery, however the reports are considered according to the time course at which tissue and electrophysiologic function was assessed following irradiation. Radiation sources are generally described as γ-radiation, β-radiation, proton beam and heavy (Carbon) ion beam. It is appreciated that the different forms of radiation have different properties and different energies meaning that their effects on tissue are not comparable but the purpose is to review patterns of damage to cardiac tissue and learn from this.

### Acute effect of cSBRT on myocardium

**Figure 1:**
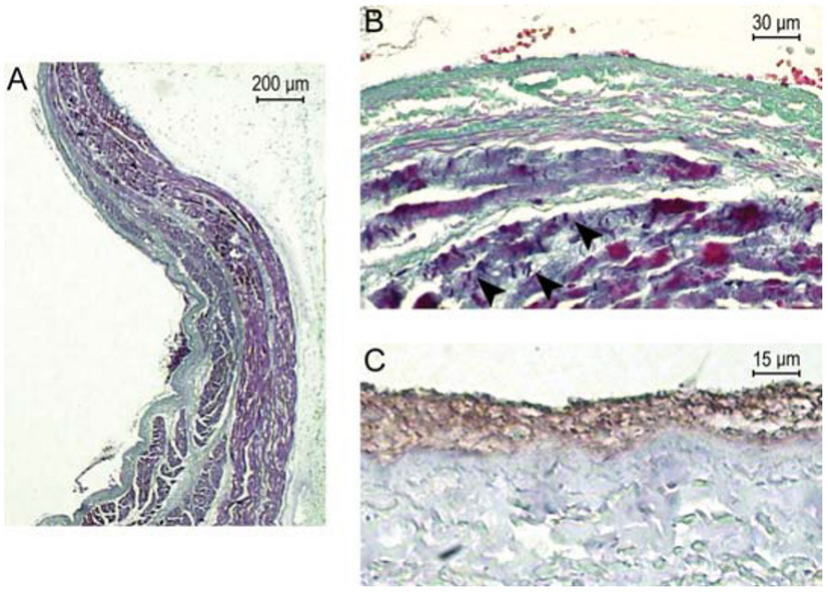
Reproduced with permission from Pérez-Castellano et al. (16). Acute pathological effects of PV β-radiation. This pig was euthanized just after radiation. **(A)** Longitudinal section of the right PV at the level of the radiated area (Masson's trichrome stain). At the luminal side of the vein (left), a laminar fibrin thrombus is adhered to the intima along a large area of endothelial denudation. A thick PV sleeve (purple) can be appreciated under the PV adventitia, which gets thinner as it enters the vein. There is some collagenous connective tissue among PV sleeve fascicles. **(B)** Previous preparation at higher magnification where PV sleeve myocardiocytes can be observed displaying marked fuchsinophilia and contraction bands (arrowheads), which are signs of impending necrosis. The absence of nuclei at the intima suggests endothelial denudation. At the lumen, there are several red blood cells included in fibrin. **(C)** CD-31 stain to best appreciate the absence of endothelial cells between the fibrin thrombus (brown) and the vein wall (gray).

A small number of studies have considered the ability of RT to induce acute effects on myocardium, which may be considered as effects seen within hours of irradiation. Lehmann et al. applied heavy ion radiation to the atrio-ventricular node of Langendorff-perfused porcine hearts (15). No acute AV conduction disturbance followed 70Gy irradiation, AV prolongation (in a heart demonstrating pre-existing Mobitz II second degree AV block) followed 90Gy irradiation, and complete AV block followed 160Gy irradiation (more than seven times the most commonly reported cSBRT dose). In this study no macroscopically visible damage was seen following irradiation and histological analysis did not reveal evidence of apoptosis or necrosis, in addition there was no evidence of increased expression of protein markers of apoptosis in the irradiated area. Hypereosinophilia was noted in the in the field irradiated with 160 Gy. Phosphorylated histone 2AX (a marker of double stranded DNA damage) was strongly positive in the irradiated region. Pérez-Castellano et al. delivered β-radiation (high-energy electrons) at a dose of 60Gy through a balloon catheter within the pulmonary vein (PV) trunk of *in-vivo* porcine hearts (16). Acute histological assessment of the acutely irradiated PV sleeve demonstrated endothelial damage, disruption of the elastic intima and myocardial sleeve necrosis. PV isolation was not achieved in this experiment. An example of the acute effects of β-radiation in this experiment is shown in Figure 1 (16).

These data indicate that acute conduction block within the specialized conduction system may be achieved with extremely high doses of heavy ion radiation, and that this was achieved without evidence of tissue necrosis or apoptosis. *In-vivo*, high dose β-radiation was associated with acute endothelial damage and myocardial necrosis, without resulting in acute conduction disturbance across the irradiated atrio-venous junction. These data do indicate an important difference between the response of tissue to RT compared to that of RF energy, following which electrophysiologic effects on both myocardial tissue and specialized conduction system tissue are seen acutely.

### Chronic effect of cSBRT on myocardium

Sharma et al. considered the impact of γ-irradiation on porcine atrial structures (17). Tissue was examined at various times (25 to 196 days) after exposure to 32–80Gy doses and demonstrated transmural loss of myocyte architecture and increased fibrin, inflammatory changes (monocyte infiltration) and discrete areas of transmural fibrosis.

Amino et al. studied the effect of heavy ion irradiation on recently infarcted leporine ventricular myocardium (18). Myocardium was irradiated two weeks following experimental MI and tissue assessment was undertaken two weeks post tissue irradiation. Compared to control animals, there was no increase in the amount of fibrosis identified. Immunohistochemistry demonstrated a 76% increase in expression of the gap junction protein Connexin-43 (Cx43) which was observed in the peri-infarct zone and remote healthy tissue, as well as changes in the distribution pattern, such that lateralization of Cx43 was observed. The group subsequently demonstrated that the increased expression of Cx43 was the result of myocardial (as opposed to fibroblast) expression and that the increased expression was likely to reflect functional gap junctions (19).

At 3 months following 25–55Gy γ-irradiation of the porcine AV node, macroscopically visible fibrosis was evident and corresponding dense fibrosis evident microscopically, with lesion volume demonstrating a strong radiation dose-response relationship (20). Following 35–40Gy γ-irradiation of the porcine AV node associated with complete AV block, loss of cellular architecture, necrosis and extensive fibrin deposition as well as fibrosis within the AV node was demonstrated (21). At 3 months following proton beam irradiation of the porcine AV node at doses of 40 – 55Gy, Suzuki et al. demonstrated heterogeneous lesions comprising mixed fibrotic changes and necrotic tissue at the targeted region, in a study in which varying degress of AV block were induced (22).

Following 60Gy β-radiation delivery 2–3 months prior in a porcine atrial model, mild neointimal hyperplasia was seen, the elastic intima was thickened and fibrosis of the PV sleeve was seen (16). There was evidence of necrosis and vacuolization in remaining myocardial tissue and inflammatory changes (mononuclear leukocyte infiltration). In addition, some calcification was noted.

In 25–50Gy β-radiation delivered *via* an intra-cardiac catheter created bidirectional CTI conduction block in a canine model (23). At 3 months, the tissue demonstrated endothelial thickening but no disruption. Transmural loss of myocyte architecture was seen, along with markers of cell death (myolysis and vacuolization) and well demarcated, transmural fibrosis.

Blanck et al. undertook a dose-finding study to assess the effect of 17.5–35Gy doses at 6 months following γ-irradiation of porcine right superior PV antrum (24). Following 17.5Gy, fatty tissue necrosis was seen, without significant fibrosis. Fatty tissue necrosis was also seen following doses of 20Gy and above, and in addition fibrosis was present, which demonstrated a dose-dependent intensity and reached transmurality following 35Gy. Subsequent work from the same group undertook a dose finding and feasibility study, this time using doses 22.5–40Gy applied to the porcine left atrial-PV junction. In this study, fatty tissue necrosis was seen following doses of 22.5Gy and higher, and fibrosis was seen following doses above 30Gy. Complete circumferential fibrosis was seen following a 40Gy dose. Of note, in this study 37.5Gy was associated with a fatal broncho-mediastinal fistula and 40Gy was associated with complete AV block secondary to AV node fibrosis (demonstrated histologically) (25). Zei et al. have also demonstrated the induction of circumferential transmural fibrosis at the right superior PV antrum when examined at 6 months following 25 and 35Gy γ-irradiation. In these specimens, there was evidence of persistent myocyte necrosis with other markers of cell damage (vacuolar degeneration with pyknotic nuclei), which was more marked in the subject receiving 35Gy compared to the one receiving 25Gy (26). Mild persistent chronic inflammation and minimal hemorrhage within the lesions was noted. In addition, this study specifically identified severe vasculitis with medial destruction, fibrinoid necrosis and luminal thrombi within the intramyocardial vessels.

Suzuki et al. considered the time course of the development of proton beam induced radiation changes in healthy porcine ventricular myocardium (22). At 12 weeks after irradiation, homogeneous necrotic tissue was seen in the lesion core and necrotic tissue and hemorrhage in the lesion border. At subsequent time points assessed (16-, 24-, and 40-weeks post irradiation), the fibrotic changes became predominant. In addition, immunohistochemical analysis demonstrated the time course of markers of apoptosis following irradiation. Caspase-3 was identified at 12 and 16 weeks but was no longer seen at 24 weeks.

**Figure 2:**
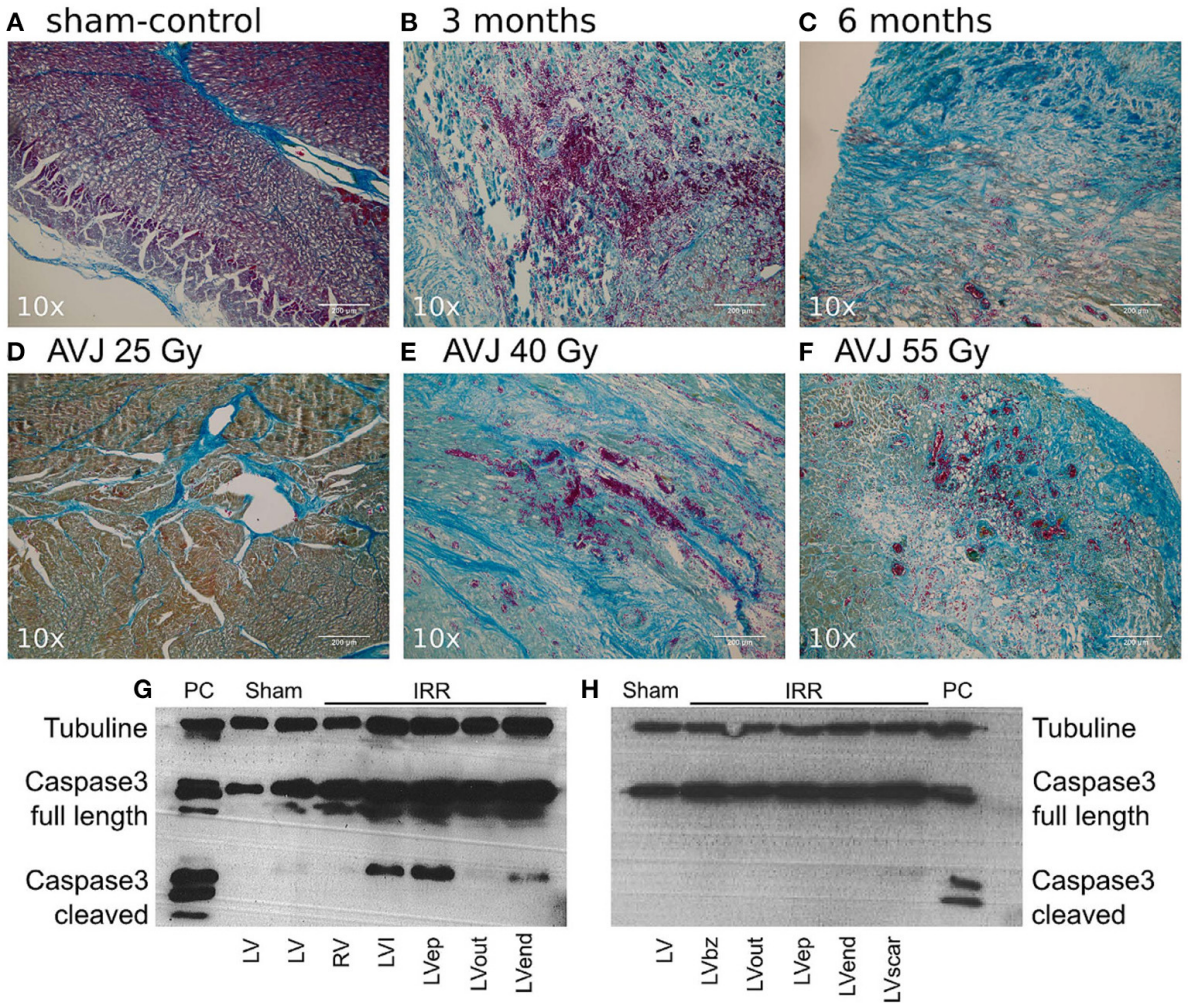
(Reproduced from Lehmann et al. (27) under Creative Commons CC YB License) Mallory Trichrome Staining of Ablation Lesions, and Apoptosis Outcomes. **(A)** Sham-control **(B)** Target tissue 3 month after 40 Gy carbon ion irradiation with marked hemorrhage, inflammation, and early stage fibrosis, **(C)** Target tissue 6 months after carbon ion irradiation, showing later stage fibrosis. **(D–F)** Comparison of myocardial lesion outcomes for 25, 40, and 55 Gy of carbon ions 6 months after irradiation for the atrioventricular junction ablation group. **(G)** Western blot for cleaved caspase-3 a marker for apoptosis; signals for cleaved caspase-3 were positive in myocardium 3 months after irradiation, whereas no signals were observed 6 months after irradiation **(H)**. Bz, borderzone; ep, Epicardium; IRR, Irradiated tissue; LV, Left ventricle; PC, positive control; HaCaT (Lysats of HaCaT cells 5 days after irradiation with 10 Gy of X-ray), I, infield; Out, Outfield; RV, Right ventricle.

Following heavy ion beam irradiation with 40Gy radiation, previously healthy porcine ventricular tissue was examined at 3 and 6 months. Targeted myocardium demonstrated hemorrhage, inflammation and early fibrosis at 3 months. In addition, caspase-3, a marker of cellular apoptosis, was present at 3 months. At 6 months, there was less marked hemorrhage and inflammation with marked fibrosis and myocyte disarray, and markers for caspase-3 were negative by this point (27). Examples of ventricular myocardial histological response to irradiation is show in Figure 2 [reproduced from Lehman et al. (27)].

Chang et al. assessed canine left atrial tissue at 6-weeks and 4-months following 33Gy γ-radiation delivered to a target area encompassing the pulmonary veins and posterior left atrial wall (28). Tissue was examined under light microscopy following Hematoxylin and eosin and Masson's trichrome staining. Immunohistochemical analysis for Cx43 was also undertaken. No gross lesions were appreciated at 6-weeks, however vacuolization, diffuse hemorrhage and inflammatory cell infiltration were appreciated. In addition, dilated capillaries were observed. At 4-months, atrial tissue demonstrated massive hemorrhage and extensive interstitial fibrosis. Of note, in this study a border-zone around the margin of the targeted area was appreciated which demonstrated a mixture of viable myocytes and fibrosis amongst significant hemorrhage. In this study, at 4-months Cx43 expression was downregulated at 4-months following 33 Gy γ-radiation.

Dose dependent upregulation of Cx43 was demonstrated up to a year following heavy ion irradiation using doses between 10–15Gy in healthy leporine ventricular myocardium (but not at 5Gy doses), without evidence of myocardial fibrosis (19).

### Clinical data

**Figure 3:**
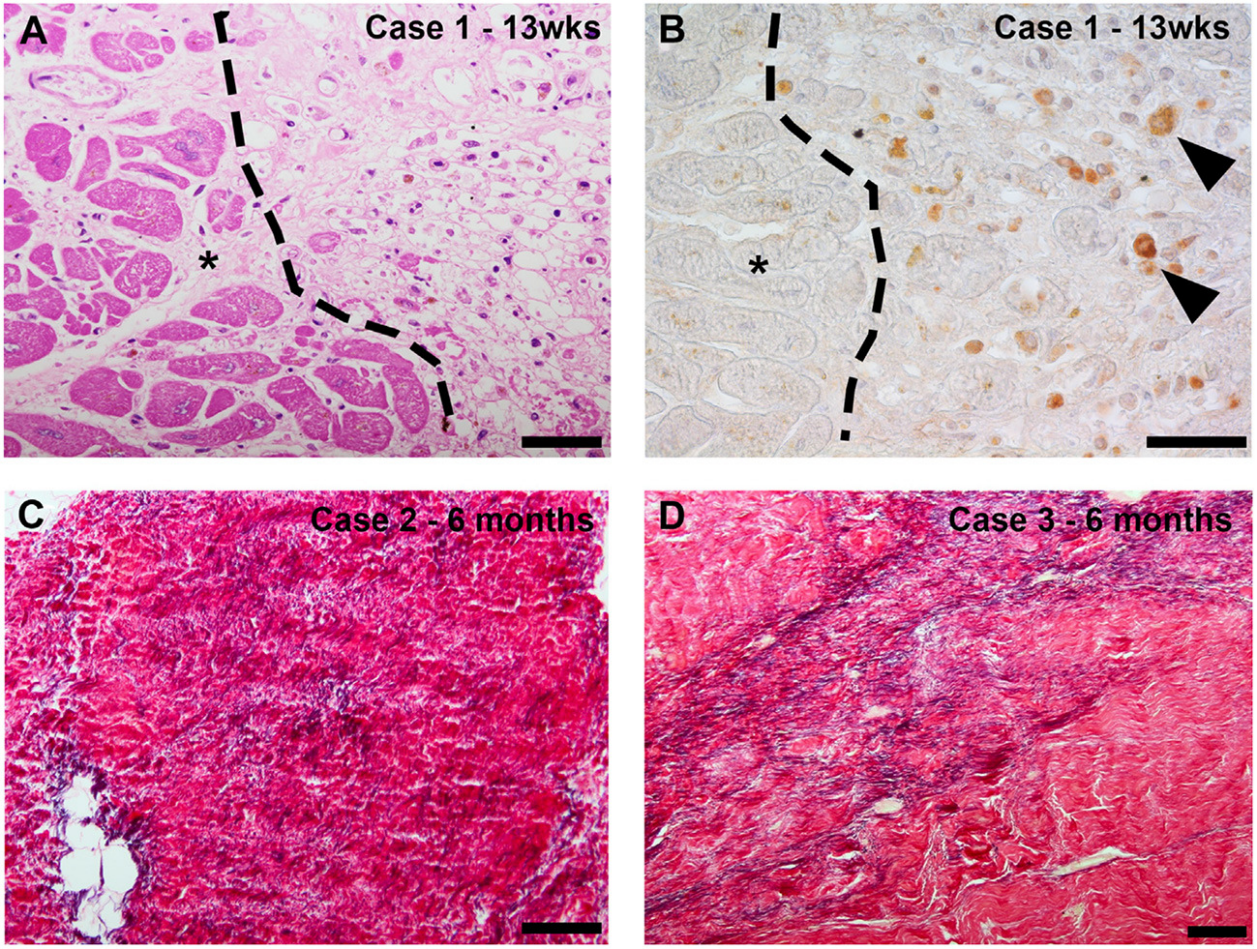
(Reproduced with permission from Kautzner et al. (32)–case numbers and details are described in referenced article): Case 1 (13 weeks after stereotactic body radiotherapy [SBRT]): routine histologic evaluation revealed myocytolysis corresponding to a previously irradiated region with a sharp transition to viable myocardium (*) **(A)**. Detection of active caspase-3 as a marker of apoptosis revealed intracellular immunoreactivity in the cytoplasm of rounded cells that morphologically corresponded to macrophages. * marks viable myocardium **(B)**. Case 2 (6 months after SBRT): histologic staining of the irradiated area revealed fibrotic region containing densely packed fibers, including a relatively high elastic component **(C)**. Case 3 (9 months after SBRT): histologic staining of the irradiated region revealed myocardial scarring with marked elastosis **(D)**.

Data from clinical specimens has been reported from a small number of cases at various time points following RT exposure. In all cases, the substrate has been targeted with 25Gy γ-radiation. In the first series of clinical cSBRT, one patient died from a stroke 3 weeks post cSBRT and histological analysis of this patient's heart was presented (29). This patient had an ischemic cardiomyopathy and note was made of prominent ectatic blood vessels at the interface of dense scar and normal tissue, without evidence of an acute vasculitis or edema. No inflammation, hemorrhage or necrosis was observed. The timing of the sample relative the cSBRT raise the possibility that the scar observed in this case was exclusively the result of the underlying cardiomyopathy. It is not clear if the observed vascular changes were related to the cardiomyopathy or represented a reaction to the RT. Krug et al. reported the histological findings from a patient with dilated mixed ischemic and non-ischemic cardiomyopathy who died 57 days post cSBRT (30). This patient underwent cSBRT for electrical storm. Within days of cSBRT, ventricular arrhythmias reduced to the point of allowing tapering of AADs, although these were subsequently re-introduced. The patient subsequently died from cardiac circulatory failure in the context of pulmonary embolus and infection. Histological analysis of the patient's heart demonstrated extensive areas of myocardial fibrosis, without clear differences between the treated and untreated regions. This report is notable for demonstration of a clear anti-arrhythmic effect of cSBRT without evidence of fibrosis formation at around 2 months post cSBRT in the context of a mixed cardiomyopathy. Kiani et al. presented the first series of histopathological specimens from patients treated with cSBRT who subsequently underwent cardiac transplantation (31). This series included 4 patients with NICM at 12–250 days post cSBRT, including one patient who had undergone 2 cSBRT treatments and included histological and ultra-structural assessments. A variety of histopathological changes were seen in the different specimens. Three cases (examined at 12, 250 and 211 days post cSBRT) demonstrated central myocyte drop out, indicating myocardial necrosis, surrounded by a rim of fibrosis. One specimen (12 days post cSBRT) demonstrated subendocardial fibrosis only without the central liquefaction noted in the other specimens. At day 12 post cSBRT, hemorrhage was seen in one sample but not the other. In addition, vascular changes were noted, including myointimal thickening and endothelial damage were seen. At 12 days post cSBRT, electron microscopy of specimens from 2 patients showed degenerative changes in myocytes. These changes included irregular and convoluted intercalated disc regions, contractile elements were absent and myofibrils were disrupted and disorganized. Mitochondria were edematous and demonstrated loss of cisternae. These observations at 12 days post cSBRT indicate acute RT induced cellular injury. Of note, there was evidence of RT-induced fibrosis in all samples, including 2 obtained at 12 days post cSBRT. Kautzner et al. assessed specimens obtained from patients who died at 3, 6 and 9 months following cSBRT (32). The substrate in this group was NICM in the patient who died at 3 months and ICM in the patients who died at 6 and 9 months. Images from this study are reproduced in Figure 3 demonstrating these histologic changes (32). On review of the pathologic specimens they identified evidence of myocytolysis at 3 months, with progressive development of fibrosis at the subsequent time points. In each specimen they noted sharp transition to viable myocardium at the edge of the targeted area. Intracellular immunoreactivity to caspase-3 was assessed in each case, and found to be positive at 3 months, less strongly positive at 6 months and absent at 9 months. These data indicate apoptosis plays a role in the early response to RT in human myocardium. Zhang et al. reported histological data from four patients at 17, 209, 251, and 478 days post cSBRT. In this report myocardium was stained with Masson's trichrome and fibrosis quantitatively assessed. In these specimens, there were only minor differences in degree of fibrosis in the RT targeted region when compared to matched untreated areas (receiving <5Gy), and these regions demonstrated significantly less fibrosis than seen in areas targeted with catheter ablation. Additional human data comes from indirect assessment of cSBRT treated myocardium in which repeat electro-anatomic mapping (EAM) followed by catheter ablation was undertaken. Gianni et al. reported 3 cases in which repeat EAM at between 6 and 12 months demonstrated preserved viability within the treated region at this time point. These data support the inference that across the time frames assessed, the anti-arrhythmic effect demonstrated in these patients was not attributable to fibrosis alone. When interpreting clinical data, it is acknowledged that in general significant heterogeneity exists between the substrate and time point at which specimens were assessed, and differentiation between substrate progression and effects of cSBRT are on occasion difficult to differentiate. Despite these caveats, dense transmural fibrosis has not been consistently identified even in the late phase following RT in human specimens following 25Gy cSBRT, despite clear demonstration of a late anti-arrhythmic effect. These observations support the concept that fibrosis alone cannot explain the clinical timeline and magnitude of reduced VT burden observed after RT. Further clinical data will be important in establishing the relative contributions of a late fibrotic effect and other anti-arrhythmic mechanisms to the efficacy of cSBRT.

### Functional effect of cSBRT

A number of studies have assessed the feasibility of using radiation to achieve conduction block in the specialized conduction system of the heart, with the AV node commonly chosen as the target. Acute AV conduction block in Langendorff perfused porcine hearts was induced with very high dose heavy ion beam irradiation (15). Heavy ion beam irradiation of the porcine AV node *in-vivo* resulted in AV block following doses of 40Gy and 55Gy, but not following 25Gy (27). This developed up to 17 weeks following irradiation. The same group have demonstrated induction of chronic AV block with γ-irradiation (20). In this study, doses of 25Gy and upwards resulted in complete AV block, which appeared at a median time of 11.2 weeks after irradiation. Refaat et al. applied γ-radiation at doses of 35–40Gy to the porcine AV node and observed complete AV block in all subjects, which appeared between 2- and 7-months following irradiation (21). Suzuki et al. induced complete AV block following *in-vivo* irradiation of the porcine AV node with proton beam radiation at doses of 40Gy and 55Gy, as well 2:1 AV block in one subject receiving 40Gy irradiation (22).

Other studies have considered the possibility of using external radiation to target common atrial locations that would be relevant to treating common atrial arrhythmias. 40Gy γ-irradiation of the CTI in a porcine model was associated with bidirectional conduction block at 30-days post irradiation, while conduction block was not observed with doses of 25Gy. At other dose levels conduction slowing across the CTI was demonstrated without conduction block (17). In the same study complete AV block was induced following 70Gy γ-irradiation and diminished amplitude of left atrial potentials at 35 days following irradiation with dose between 38–80Gy. Guerra et al. demonstrated bidirectional CTI conduction block in a canine model following delivery of 25–50Gy β-radiation delivered via an intra-cardiac catheter, which was demonstrated at between one- and four-weeks post irradiation.

β-radiation at a dose of 60Gy through a balloon catheter within the pulmonary vein (PV) trunk resulted in diminished PV amplitude and elevated pacing threshold without conduction block in or out of the vein at 81 days post irradiation (16).

At 6 months following 17.5–35Gy γ-irradiation of the right superior PV, pulmonary vein isolation was not achieved in a porcine model, likely due to incomplete circumferential transmural ablation in this study (24). However, in a subsequent study circumferential transmural fibrosis was achieved with a dose of 40Gy, which resulted in electrical isolation of the right superior PV (25). Of note, in addition to PV isolation, there was AV node fibrosis resulting in complete AV block. In those animals in whom PV isolation was not observed, there was a dose-dependent reduction in PV electrogram amplitude. Zei et al. demonstrated electrical right superior pulmonary vein isolation at 3–6 months following γ-irradiation at doses of 25–35 Gy, electrical isolation in some subjects (with partial effect in the remainder) following 20Gy and no or partial effect in subjects receiving 15Gy. Combined pulmonary vein and posterior left atrial wall isolation was achieved in 3 of 4 animals at 4-months following 33Gy γ-irradiation in a canine model. Conduction block was not apparent in two animals assessed at 6-weeks post cSBRT using the same protocol (28).

Previously healthy porcine ventricular myocardium underwent *in-vivo* proton beam irradiation and systolic function was assessed over time (34). At 16 weeks, fibrosis was seen in regions receiving 20–30Gy doses, and in the lesions core, which received >30Gy necrotic tissue was seen, with vacuolar degeneration and myocytolysis present. This study demonstrated a dose dependent effect on LV systolic function and LV end-diastolic volume–a greater volume of myocaridum treated with cSBRT in a range to cause necrosis and subsequent fibrosis was associated with a reduction in LV systolic function. Dusi et al. provide further evidence of the potential impact of cSBRT on myocardial function in the first reported case of proton-beam radiation cSBRT for VA. In this report there was a local decrease in longitudinal strain noted in the targeted region at 1 month post cSBRT, supporting an early local mechanical effect of cSBRT, as well as effective suppression of previously incessant VA (35). These observations are of particular relevance when consideration is given to the potential for a substrate-based delivery of cSBRT, as recently reported by Qian et al. (36), during which extensive regions may be considered for targeting. The demonstrable impact on LV function following delivery of cSBRT to a large anatomic area support the paramount importance of a precise target definition and focused energy delivery for cSBRT.

Data from a small number of patients has been reported assessing surface ECG data from patients undergoing cSBRT. Zhang et al. report a non-significant trend toward QRS shortening among 19 patients who underwent cSBRT, including 4 of whom demonstrated a robust 25ms shortening of the QRS duration at 6-weeks post cSBRT (33). A reduction in late potentials on high-resolution signal averaged ECG from a single patient was also noted by Amino et al. at 6-weeks post-cSBRT (37), an effect which persisted out to 6-months. The mechanism underlying these observations, as well as their clinical relevance remains uncertain. These data are consistent with reduction in volume of late activated myocardium, changes in myocardial CV or a combination of these and other mechanisms.

### cSBRT in experimental models of ventricular substrate

In a leporine model of recent MI, heavy ion beam irradiation resulted in changes in ventricular conduction velocity in both control tissue and in the peri-infarct border zone at 2 weeks following irradiation. These may be summarized as demonstrating an increase in transverse CV following irradiation in both control and peri-infarct tissue, and in addition an increase in longitudinal CV in peri-infarct tissue, reversing the CV slowing seen in peri-infarct tissue following MI without irradiation. Therefore, as well affecting total activation time of the ventricle, irradiation affected the ventricular anisotropic conduction properties in both healthy and peri-infarct tissue. In addition, refractoriness was prolonged in healthy tissue following irradiation. In this study, irradiation resulted in an overall decrease in the susceptibility to inducible ventricular arrhythmias (18).

In a canine model of recent MI, 15Gy of heavy ion beam irradiation resulted in reversal of MI-induced diminished expression of Cx43 seen in the peri-infarct border zone at 1 year. Of note, this was also associated with reduced surface ECG evidence of delayed ventricular activation, reduced susceptibility to induced ventricular arrhythmias as well as greater recovery of LV systolic function (as assessed by echocardiographic fractional shortening) without effect on diastolic function at 1 year post irradiation (38).

At 4 weeks following experimental MI, proton beam irradiation was applied to porcine ventricular tissue (targeting CMR identified infarct) at doses of 30–40Gy (39). In this study, no differences in the histological appearance of tissue from treated and untreated areas was noted at 8 weeks. At 15 weeks, progressive interstitial fibrosis was seen in the peri-infarct zone as well as in targeted healthy tissue, along with signs of inflammation (mononuclear cells). By 6 months, tissue in the peri-infarct zone receiving >30Gy was largely homogenized, with widespread myocytolysis and vacuolar degeneration as well as fibrosis. A reduced proportion of surviving cardiomyocytes and an increase in the proportion of collagen was demonstrated at 6 months post irradiation in treated areas when compared to untreated peri-infarct tissue. Of note, with radiation constrained to the scar in this model there was no adverse impact on LV systolic function.

**Figure 4:**
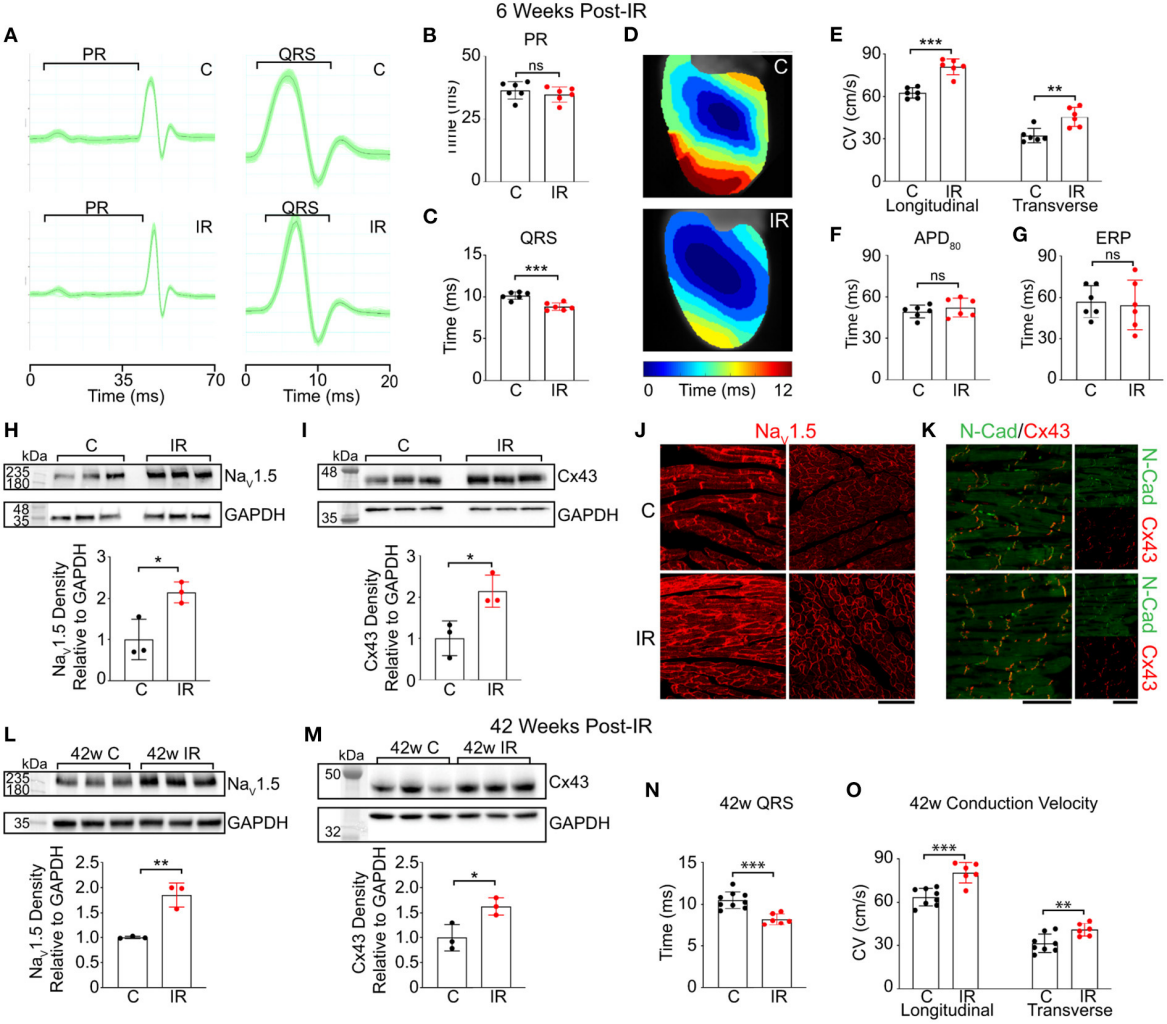
Reproduced from Zhang et al. (33) under Creative Commons Attribution 4.0 International License (http://creativecommons.org/licenses/by/4.0/) Cardiac RT persistently increases adult murine ventricular conduction. **(A)** Representative ECGs from control (top) and IR mice (bottom) highlighting PR and QRS intervals. **(B,C)** Effect of radiation on PR (*P* = 0.39) and QRS (**P = 0.00043) intervals in control (black) vs. IR (red) mice (*n* = 6 biologically independent animals per condition). **(D)** Representative ventricular activation maps from control and IR mice. Scale bars =3 mm. **(E)** Effect of RT on ventricular CV (*n* = 6 biologically independent animals per condition; ***P = 0.00005; **P = 0.0034). **(F,G)** APD80 (*P* = 0.39) and ERP (*P* = 0.78) in control versus IR mice (*n* = 6 biologically independent animals per condition). **(H,I)** Western blots of NaV1.5 (*P = 0.022) and Cx43 (*P = 0.025) in control and IR ventricles (*n* = 3 biologically independent samples per condition). **(J)** Immunostaining of control (top) and IR (bottom) myocardium for NaV1.5 and **(K)** co-stained for Cx43 (red) and N-Cadherin (green). Scale bars= 70 μm. Experiment was replicated 3 times in biologically independent specimens and produced similar results. **(L,M)** Western blots of NaV1.5 (**P = 0.0034) and Cx43 (*P = 0.027) after 42 weeks post-IR (*n* = 3 biologically independent samples per condition). **(N)** Effect of RT on QRS intervals in control vs. IR mice after 42 weeks (***P = 0.00027; C, *n* = 9; IR, *n* = 6). **(O)** Left ventricular CVs in control (*n* = 9 biologically independent animals) vs. IR (n = 6 biologically independent animals) mice at 42 weeks post-IR (***P = 0.00042; **P = 0.0092). All *P* values determined by two-way unpaired *t* test. Bar graphs are represented as mean ± SD.

Zhang et al. considered the early effects of RT on conduction velocity (33). At 2 weeks post experimental MI in a murine model, 25Gy γ-radiation was applied to the whole heart. At 6 weeks post irradiation myocardial CV was assessed using optical mapping, and a significant increase in both longitudinal and transverse CV was identified in both normal tissue and peri-infarct border zone, without clear change in the CV anisotropy ratio. At this time point, no increase in fibrosis was detected on histological assessment, although immunohistochemical analysis identified an increase in voltage gated sodium channel (Na_V_1.5) and Cx43 proteins, without change in their pattern of distribution. Further data is presented using gain and loss of function knock-out mice (without infarct), to indicate that activation of the Notch signaling pathway is involved in regulation of RT induced changes in CV and protein expression, and that upregulation of Na_V_1.5 persisted out to a year following transient Notch activation, while Cx43 in this experiment was not increased at this time point. These data are sumarized in Figure 4 (33). This data, alongside the work from Amino et al. (19, 38), indicates a potential mechanism for the early anti-arrhythmic effect of cSBRT that has been consistently observed in clinical studies. There are a number of plausible mechanisms through which increasing CV could effect an anti-arrhythmic effect including changes in reentrant wavelength and conduction safety factor, but these remain speculative at present. In addition to demonstrating an increase in CV following RT, Zhang et al. used a dose-escalation approach to demonstrate that this electrophysiological response was present following single fractions doses as low as 15Gy (33). Given the potential importance of this change in CV in mediating the desired anti-arrhythmic effect, this result raises the possibility delivering an effective treatment at lower RT lower dose, with the potential for reduced toxicity if this was the case. This observation motivates further research to establish the minimum dose required to achieve the anti-arrhythmic effect of cSBRT, in order to minimize the toxicity associated with this treatment while maintaining the efficacy. Of note, both of the studies in which an acute increase in conduction velocity was observed used models of early MI, representing an important difference from the typical clinical situation of VA in the context of chronically remodeled ventricular tissue. Note is made of the improvement in LV function observed in the canine study (38) which raises the possibility of a broader effect on the healing process beyond the specific conduction properties of tissue, which may be relevant to the observations made. If this was the case, it may affect the likelihood of these observations being seen in the chronic remodeled myocardium typically treated in the clinical setting, but there is no data assessing this currently available. As yet, no clinical data regarding early changes in conduction velocity in humans has been presented and the early biological effect of RT on human myocardium remains undefined.

## Conclusion

cSBRT represents a promising non-invasive modality that has recently emerged for the treatment of refractory VA. The myocardial response to RT is complex and likely to be cell-type specific. In addition, amongst similar cell types, the radio-sensitivity of tissue may be different in healthy vs. unhealthy myocardium. Acute and sub-acute cellular changes following cSBRT including those of cellular necrosis and apoptosis have been identified in experimental and clinical reports. Vascular effects, including vasculitis and capillary thrombosis, as well as acute mitochondrial damage have been reported in the acute phase following cSBRT. These changes precede the development of myocardial fibrosis, which has most commonly been seen beyond 3 months from cSBRT. Furthermore, in several clinical reports, no discernible histopathological changes were identified in the treated myocardium despite a reduction in the arrhythmia burden of the patients following cSBRT. The cancer community are increasingly aware of radiation induced damage to normal tissue in patients receiving RT for cancers; especially dose to cardiac substructures. There are many ongoing pre-clinical and clinical studies in this area providing much more data which may give further useful insights into mechanisms of damage.

In experimental conditions, acute conduction block within specialized conduction tissue may be achieved, in some cases with doses well in excess of those that have been used to achieve arrhythmia suppression clinically. In contrast to the mechanism through which arrhythmia suppression is achieved with traditional catheter-based technologies, acute conduction block does not appear to be the mechanism underlying the early anti-arrhythmic of cSBRT. Experimental studies have indicated the possibility of acute increases in CV in experimental models of early MI and suggested that RT induced electrical reprogramming of myocardial gap junction and sodium channel expression may persist up to a year after RT exposure. The relevance of these observations to the early, and indeed late, anti-arrhythmic effects of cSBRT remain uncertain but represent an intriguing area for future investigation. Clinical data assessing the CV in human myocardium following cSBRT has not been reported. The role of late-stage fibrosis in the homogenization of arrhythmogenic myocardial substrate may represent a more familiar mechanism through which arrhythmias are inhibited, but even data confirming this are so far limited.

The structural and functional myocardial response to RT exposure through cSBRT is likely to be specific to the cell type targeted, the dose and radiation source of the RT delivered and the time-point at which tissue is assessed. There exists a large knowledge gap regarding many of the topics discussed in this review which represents an exciting opportunity for future research. As experience grows with the wider use of cSBRT, a greater understanding of the tissue response will likely develop and this may contribute to further insights into the mechanisms through which cSBRT provides acute and chronic arrhythmia suppression. In the future, with the benefit of a greater understanding of the many outstanding issues discussed in this review, it is envisaged that cSBRT therapy could be planned to optimally titrate both early and late radiation effects to optimize the combined anti-arrhythmic efficacy of the treatment, minimize radiation associated toxicity and thus achieve the best patient outcomes.

## Author contributions

First draft created by JW. Critical review and significant contributions from PZ, SA, SN, MO'N, and CR. All authors contributed to the manuscript submitted and final draft reviewed, and approved.

## Funding

This research was funded in whole, or in part, by the Wellcome Trust (WT 203148/Z/16/Z). For the purpose of open access, the author has applied a CC BY public copyright licence to any Author Accepted Manuscript version arising from this submission.

## Conflict of interest

The authors declare that the research was conducted in the absence of any commercial or financial relationships that could be construed as a potential conflict of interest.

## Publisher's note

All claims expressed in this article are solely those of the authors and do not necessarily represent those of their affiliated organizations, or those of the publisher, the editors and the reviewers. Any product that may be evaluated in this article, or claim that may be made by its manufacturer, is not guaranteed or endorsed by the publisher.
